# Lipid Nanocapsules Loaded with Rhenium-188 Reduce Tumor Progression in a Rat Hepatocellular Carcinoma Model

**DOI:** 10.1371/journal.pone.0016926

**Published:** 2011-03-07

**Authors:** Claire Vanpouille-Box, Franck Lacoeuille, Jérôme Roux, Christophe Aubé, Emmanuel Garcion, Nicolas Lepareur, Frédéric Oberti, Francis Bouchet, Nicolas Noiret, Etienne Garin, Jean-Pierre Benoît, Olivier Couturier, François Hindré

**Affiliations:** 1 LUNAM Université, Université d'Angers, INSERM U646, Angers, France; 2 Nuclear Medicine Department, Angers CHU, Angers, France; 3 LUNAM Université, Université d'Angers, SCAHU, UFR Medicine, Angers, France; 4 Medical Imaging Department, CRLCC Eugene Marquis, Rennes, France; 5 European University of Brittany, Rennes, France; 6 Radiology Department, Angers CHU, Angers, France; 7 LUNAM Université, Université d'Angers, Laboratory HIFI, UPRES EA3589, Angers, France; 8 UMR CNRS 6226, ENSCR, Rennes, France; Université de Technologie de Compiègne, France

## Abstract

**Background:**

Due to their nanometric scale (50 nm) along with their biomimetic properties, lipid nanocapsules loaded with Rhenium-188 (LNC^188^Re-SSS) constitute a promising radiopharmaceutical carrier for hepatocellular carcinoma treatment as its size may improve tumor penetration in comparison with microspheres devices. This study was conducted to confirm the feasibility and to assess the efficacy of internal radiation with LNC^188^Re-SSS in a chemically induced hepatocellular carcinoma rat model.

**Methodology/Principal Findings:**

Animals were treated with an injection of LNC^188^Re-SSS (80 MBq or 120 MBq). The treated animals (80 MBq, n = 12; 120 MBq, n = 11) were compared with sham (n = 12), blank LNC (n = 7) and ^188^Re-perrhenate (n = 4) animals. The evaluation criteria included rat survival, tumor volume assessment, and vascular endothelial growth factor quantification. Following treatment with LNC^188^Re-SSS (80 MBq) therapeutic efficiency was demonstrated by an increase in the median survival from 54 to 107% compared with control groups with up to 7 long-term survivors in the LNC^188^Re-SSS group. Decreased vascular endothelial growth factor expression in the treated rats could indicate alterations in the angiogenesis process.

**Conclusions/Significance:**

Overall, these results demonstrate that internal radiation with LNC^188^Re-SSS is a promising new strategy for hepatocellular carcinoma treatment.

## Introduction

Hepatocellular carcinoma (HCC) is the fifth most common malignant tumor worldwide. The prognosis of HCC remains extremely poor, and a curative treatment (liver transplantation, surgical resection, and radiofrequency ablation) can only be carried out in approximately 25% to 30% of cases [1]. The use of conventional external beam radiation therapy in HCC treatment has been limited by the low radiation tolerance of the cirrhotic liver that often resulted in radiation-induced liver disease (RILD) [2]. Selective internal radiotherapy (SIRT) aims to deliver high tumoricidal doses while limiting the development of RILD. This locoregional strategy is defined as the infusion of radioactive carrier including microsphere of Yttrium-90, Iodine-131 iodized oil or similar agent into the hepatic artery [3]. Currently, ^90^Y-microspheres are the most SIRT technique used. Given the hypervascularity of HCC, ^90^Y-microspheres injected into the hepatic artery will spread throughout the liver or confined to certain areas, where they can stop blood supply of the tumor by the embolisation process.

Progress in pharmaceutical research field has been exploited in the design of tumor-targeting nanoscale vectors able to deliver radionuclides. Among them, lipid nanocapsules (LNC), a nanovector with biomimetic properties [4], appear to be a useful therapeutic option for HCC treatment. Composed of a liquid lipidic core surrounded by a tensioactive shell, LNC lead to the encapsulation of a lipophilic complex of Rhenium-188 (γ = 155 keV; β^-^ = 2 MeV; T_1/2_ = 16.9 h). The formulation, based on a fully automated phase-inversion process, is simple and results in nanoparticles solution presenting a mean diameter between 20 and 100 nm, depending on the quantity of excipients. The nanometric scale of LNC^188^Re-SSS (50 nm) could be highly advantageous, as LNCs may penetrate more deeply inside the tumor blood vessels, the mean diameter of microsphere devices are varying between 20 to 500 µm. Moreover, enhanced permeability retention effect (EPR), the main strategy for the delivery of nanoparticulate systems, may improve therapeutic efficiency. Indeed, it has been shown that small particles can passively cross the sinusoidal endothelium of the liver through fenestrations with a size of approximately a few hundred nanometers [5], [6].

We report a study of LNC^188^Re-SSS as a new radiopharmaceutical carrier for internal radiotherapy of rats presenting hepatocellular carcinoma induced by diethylnitrosamine. No early mortality and no intolerance following LNC^188^Re-SSS intra-arterial injection were observed. Our results provide evidences of therapeutic efficiency of LNC^188^Re-SSS with a reduction in tumor progression which could be combinated with an altered angiogenesis process as indicated by VEGF quantifications in plasmatic samples in a rat HCC model.

## Methods

### Ethics Statement

This study was carried out in strict accordance with the French Minister of Agriculture and the European Communities Council Directive of 24 November 1986 (86/609/EEC). The protocol was approved by the Committee on the Ethics of Animal Experiments of the “Pays de la Loire” (Permit Number: CEEA.2009.6). All surgery was performed under ketamine/xylazine anesthesia, and all efforts were made to minimize suffering.

### Materials

Lipoïd® S75-3 (soybean lecithin with 69% of phosphatidylcholine) and Solutol® HS15 (a mixture of polyethylene glycol 660 and polyethylene glycol 660 hydroxystearate) were kindly donated by Lipoïd Gmbh. (Ludwigshafen, Germany) and BASF (Ludwigshafen, Germany), respectively. NaCl and dichloromethane were provided by Sigma (St-Quentin, Fallavier, France). Deionized water was obtained from a Milli-Q plus system (Millipore, Paris, France). Lipophilic Labrafac® CC (caprylic-capric acid triglycerides) was provided by Gattefosse S.A. (Saint-Priest, France).

### Preparation of the ^188^Re-SSS complex

^188^Re as carrier-free Na [^188^ReO_4_^-^] in a physiological solution was obtained by saline elution of a ^188^W/^188^Re generator (*Institut des Radioéléments* [Institute for Radioelements], Fleurus, Belgium) and then concentrated. The ^188^Re-SSS complex was prepared according to the method developed by Lepareur *et al*.[7]. In brief, the ^188^Re-SSS complex was obtained by the reaction of the ligand sodium dithiobenzoate (organic synthesis platform, Rennes, France) with a freeze-dried formulation kit containing 30 mg sodium gluconate, 30 mg ascorbic acid, 40 mg potassium oxalate, and 4 mg SnCl_2_.2H_2_O reconstituted in 0.5 mL of physiological serum. 1 110 MBq of ^188^Re-perrhenate (^188^ReO_4_^-^, in 0.5 mL) was added, and the solution was mixed for 15 minutes at room temperature. Then, 20 mg of sodium dithiobenzoate (in 0.5 mL; pH = 7) was added before being heated at 100°C for 30 minutes, which allowed for the formation of the ^188^Re-SSS complex. Due to its precipitation in aqueous media, the ^188^Re-SSS complex was extracted with dichloromethane (1 mL) and washed three times with 1 mL of deionized water. The radiochemical purity (RCP) of the complex was checked by thin-layer chromatography as the ratio of migrated radioactivity to total radioactivity. Thin-layer chromatography was carried out using silica gel 60-F_254_ alumina plates (Merck) and a solution of petroleum ether/dichloromethane (6/4; v/v) as an eluant. Radioactivity was assessed with a phosphor-imaging machine (Packard, Cyclone storage phosphor system).

### Nanocapsule formulation and characterization

The overall study was performed on 50 nm-diameter LNCs which were prepared according to a phase-inversion process described by Heurtault *et al*. [4]. In brief, 25 mg Lipoïd ® S75-3, 282 mg Solutol® HS15, 342.7 mg Labrafac®, 29.7 mg NaCl, and 987.5 mg deionized water were mixed by magnetic stirring. The ^188^Re-SSS complex extracted with dichloromethane (1 mL) was then added to the other components of the emulsion. The organic solvent was removed by being heated at 60°C for 15 minutes. Three cycles of progressive heating and cooling between 85°C and 60°C were then carried out and followed by an irreversible shock, induced by dilution with 4.16 mL of 0°C deionized water, which was added to the mixture at 70°C leading to 50 nm-lipid nanocapsules solution. Afterwards, slow magnetic stirring was applied to the suspension for 5 minutes. LNC^188^Re-SSS were dialyzed during 2 hours with deionized water at room temperature by magnetic stirring. The mean diameter and polydispersity index were then determined using a Malvern Zetasizer® Nano Serie DTS 1060 (Malvern Instruments S.A., Worcestershire, UK). The encapsulation yield was assessed with a gamma counter (Packard Auto-Gamma 5,000 series) according to the equation below
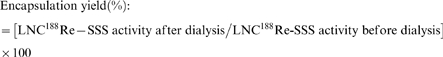



### HCC model and treatment

49 Male Wistar rats weighting 150–180 g were obtained from the animal house of the Angers University Hospital. The animals were kept in polycarbonate cages in a room with controlled temperature (20–22°C), humidity (50–70%), and light (12-hour light/dark cycles). Room air was renewed at the rate of 10 vol/hour. Tap water and food were provided *ad libitum*.

All experiments were performed on 6-week-old male Wistar rats. Hepatic carcinogenesis was induced chemically by adding diethylnitrosamine (DENA) to drinking water (100 mg/L) for 8 weeks. Each animal underwent hepatic artery catheterization on Day 10 (D10) after the end of tumor induction. Two LNC^188^Re-SSS groups were performed with one intra-hepatic artery injection of 80 MBq and 120 MBq of LNC^188^Re-SSS (LNC^188^Re-SSS – 80 MBq, n = 12; LNC^188^Re-SSS – 120 MBq, n = 11) as described in the “Hepatic artery catheterization” section below. Three control groups, the ^188^ReO_4_^-^ group (80 MBq, n = 4), the sham group (n = 12) and the blank LNC group (n = 10) were assessed with the same procedure. We set the therapeutic activity at 80 MBq and 120 MBq, corresponding to an absorbed dose to the liver of 40Gy, which proved to be effective in the treatment of human HCC [8], and of 65Gy in order to appreciate the effect of a higher dose after LNC^188^Re-SSS internal radiation.

### Hepatic artery catheterization

Hepatic artery catheterization was carried out according to the Garin *et al*. method [9]. The experiment was continued under a binocular magnifying glass. The first duodenal loop was pulled down to expose the gastroduodenal artery which was, after identification of the celiac and hepatic arteries, carefully dissected. A final distal ligature of the gastroduodenal artery was performed, and the celiac artery was clamped to temporarily stop arterial flow. After perforation using a 30 G needle, a 32 G catheter (CS-32, Bioseb, Vitrolles, France) was placed inside the gastroduodenal artery, and a volume of 400 µL of LNC^188^Re-SSS, ^188^ReO_4_^-^, or blank LNC solution was then injected. After washing the syringe with saline solution, the upstream end of the gastroduodenal artery was tied off and arterial flow was restored. Concerning the sham group, the same procedure was performed except the injection which was not realized and a final ligature of the hepatic artery. This control group led to an appreciation of the collateral revascularization which could occur after injection.

### Transaminase assessment

Blood samples were collected from the tail vein using heparinized tubes in each group at D12, D18, D24, D25, D30, D45, D55, D65, D80, D90, D105, D130, and D152 after the end of tumor induction in order to assess hepatotoxicity after treatment injection. After centrifugation at 1 000 g for 20 minutes, plasma alanine aminotransferase (ALT) and aspartate aminotransferase [10] were assessed using BioMérieux kits (Marcy l'étoile, France) according to the manufacturer's instructions.

### Dosage of plasmatic vascular endothelium growth factor (VEGF)

Blood samples were collected from the tail vein using heparinized tubes in each group at D12, D20, D40, D55, D65, D80, and D105 after the end of tumor induction. After centrifugation at 1 000 g for 20 minutes, the rat VEGF ELISA test (R&D Systems Europe, Lille, France) was immediately performed according to the manufacturer's instructions.

### Magnetic resonance imaging

Six animals from the LNC^188^Re-SSS – 80 MBq and four animals from the ^188^Re-perrhenate groups were explored by MRI in order to acquire images of the treatment-induced morphological changes at day 100 after the end of tumor induction. MRI was performed with a 1.5T Signa Excite HD device (General Electric Medical System Milwaukee, Ilinois). Rapid T2-weighted images were obtained using a fast spin echo (FSE) sequence with the following parameters: TR = 2500 s, TE = 102 s, matrix 320×320, twelve slices, thickness 3 mm, gap 0.5 mm, FOV = 15 cm, Nex = 4, acquisition time 6′30″). The following day, the animals were sacrificed for macroscopic and histopathological analyses.

### Whole-body planar γ-scintigraphy

Three animals from the LNC^188^Re-SSS – 80 MBq and three animals from the ^188^Re-perrhenate groups were explored by whole-body planar γ-scintigraphy. Static 15-minutes scintigraphy acquisitions were obtained using a gamma camera (SOPHA DSX γ camera, 155 keV±15%, 128^2^ matrix, HRLE collimator). ^188^Re-perrhenate and LNC^188^Re-SSS solutions (80 MBq) were injected into the hepatic artery, and images were taken at 1.5 h, 3 h, and 24 h. A scan projection radiograph (Scout View) was acquired on a GE lightspeed system (General Electric Medical System Milwaukee, Ilinois) and fused with planar scintigraphy of the same animal for the localization of ^188^ReO_4_^-^ and LNC^188^Re-SSS.

### Tissue distribution study

A tissue distribution study was carried out on an extra group of 24 male Wistar rats with chemically-induced HCC. They were divided into two groups: one injected with a ^188^ReO_4_^-^ solution following hepatic artery catheterization (n = 12), and the other with LNC^188^Re-SSS – 80 MBq (n = 12). In both groups, the animals were sacrificed at 1.5 h (n = 4), 3 h (n = 4), and 24 h (n = 4) post-injection. The organs were removed, washed, and weighed (blood, liver, spleen, kidneys, heart, lung, stomach, small intestine, large intestine, bladder, bone, muscle, brain, tail, and carcass). The content activity of each organ was determined using a gamma counter (Packard Auto-Gamma 5,000 series).

### Statistical analysis

The Kaplan-Meier method was carried out to plot animal survival. Statistical significance was assessed using the log-rank test (Mantel-Cox Test). StatView software was used for this purpose, and the tests were considered significant at p values less than 0.05. The different groups were compared in terms of survival time, increase in median survival time (IMST %), maximal survival time, and long-term survivors (rats were considered to be long-term survivors if they survived twice the median survival time of control groups [11]).

## Results

### Encapsulation of the ^188^Re-SSS complex in LNCs

The ^188^Re-SSS complex presents a good purity as satisfactory RCP of more than 98% was obtained. Physico-chemical characteristics of the LNC are provided in Table 1. Blank LNCs were measured at 50.7±5.7 nm. ^188^Re-SSS entrapping did not change the characteristics of nanoparticles with a size of 49.7±2.7 nm. Blank LNC and LNC^188^Re-SSS solutions were monodispersed with a polydispersity index of 0.05 (Table 1). The encapsulation yield was approximately 97.9%, 97.7% and 97.5% after 2 h, 24 h and 48 h of dialysis, against phosphate buffer solution (pH = 7.4), respectively; therefore, ^188^Re-SSS release of 2.5% observed at 48 h can be neglected.

**Table 1:** Physico-chemical characteristics of blank and ^188^Re-SSS LNCs.

	Mean particle size	Polydispersity index	Encapsulation yield (%)
Blank LNC	50.7±5.7	0.05±0.02	-
LNC^188^Re-SSS	49.7±2.7	0.05±0.03	97.9±0.2

### Biodistribution study

**Figure 1:**
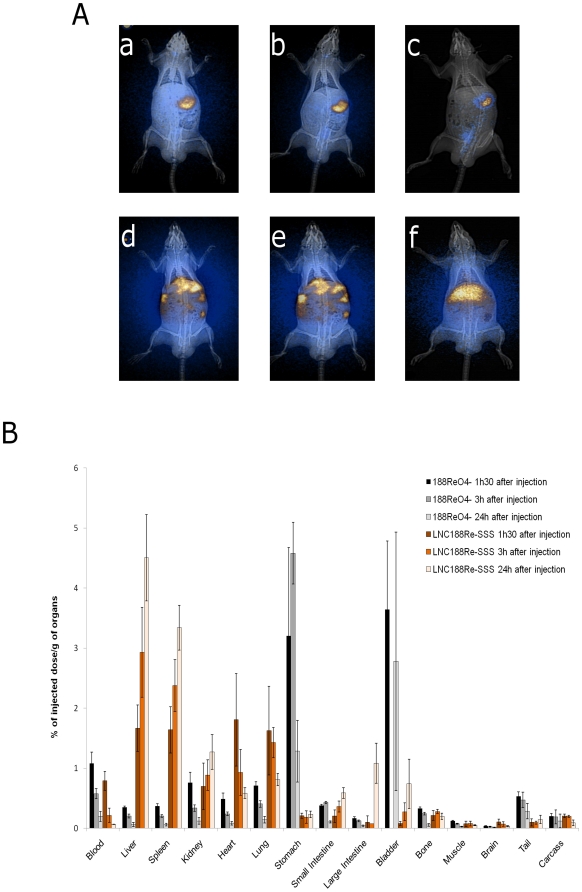
TEP images after the catheterization of the hepatic artery and tissue distributions. **a**: A, B, and C: TEP images after 1.5 h, 3 h, and 24 h of the ^188^Re-perrhenate solution; D, E, and F: TEP images after 1.5 h, 3 h, and 24 h of the LNC^188^Re-SSS solution **b**: Organ biodistribution of ^188^ReO_4_^-^ (n = 12) and LNC^188^Re-SSS (n = 12) solutions 1.5 h, 3 h, and 24 h following the intra-hepatic injection. Results are expressed as a percent of injected dose per gram of organ, mean ± SD.

Planar scintigraphy led to the monitoring of the radioactivity distribution of LNC^188^Re-SSS and ^188^ReO_4_^-^ following hepatic artery injection (Figure 1a). Results highlighted a liver uptake following LNC^188^Re-SSS injections and a stomach uptake after ^188^ReO_4_^-^ injection up to 24 hours.

LNC^188^Re-SSS were essentially confined to the liver, with an increased uptake up to 24 h (1.5 h post-injection: 1.67±0.39%ID/g; 3 h post-injection: 2.93±0.75%ID/g; 24 h post-injection: 4.51±0.72%ID/g) in correlation with an increased of the blood clearance (1.5 h post-injection: 0.79±0.16%ID/g; 3 h post-injection: 0.21±0.12%ID/g; 24 h post-injection: 0.06±0.01%ID/g).

Contrary, the tissue distribution study of ^188^Re-perrhenate solution revealed a rapid stomach uptake (1.5 h post-injection: 3.21±1.47%ID/g; 3 h post-injection: 4.58±0.85%ID/g; 24 h post-injection: 1.28±0.51%ID/g).

### Survival study

The descriptive and statistical data from the survival study are summarized in Table 2.

**Table 2:** Descriptive and statistical data from the survival study.

	Median survival time (days)	IMST (%)		Max survival time (days)	Min survival time (days)	Long-term survivors		*p* values versus sham	*p* values versus blank LNC	*p* values versus ^188^ReO_4_^-^	*p* values versus LNC^188^Re-SSS80 MBq	*p* values versus LNC^188^Re-SSS120 MBq
		Sham	^188^ReO4^-^			Sham	^188^ReO4^-^					
**LNC^188^Re-SSS - 80 MBq** (n = 12)	118±27	54	107	154	72	1	7	0.0139*	0.0118^£^	0.0102 ^$^	-	0.4431
**LNC^188^Re-SSS -120 MBq** (n = 11)	97±18	26	70	140	74	0	2	0.0367	0.0236^£^	0.0219^$^	0.4431	-
**Sham** (n = 12)	77±19	6	34	105	49	0	0	-	0.2440	0.4247	0.0139^§^	0.0367
**Blank LNC** (n = 7)	51±19	-34	−11	92	38	0	0	0.0749	-	0.7237	0.0118^§^	0.0236^#^
**^188^ReO_4_^-^**(n = 4)	57±24	−26	-	90	41	0	0	0.4247	0.7237	-	0.0102^§^	0.0219^#^

The increase in the median survival time (IMST%) is calculated in comparison to the sham and the ^188^Re-perrhenate groups. Comparisons of survival data using the log-rank test (Mantel-Cox test) versus sham group (*p<0.05), blank LNC (^£^ *p*<0.05), ^188^Re0_4_^-^ group (^$^ *p*<0.05), LNC^188^Re-SSS – 80 MBq (^§^ *p*<0.05), LNC^188^Re-SSS – 120 MBq (^#^ *p*<0.05).

**Figure 2:**
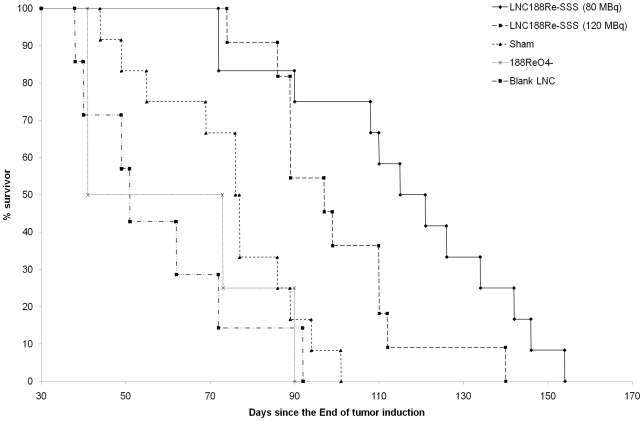
Kaplan-Meier survival curves of rats with induced HCC. On D10, rats were treated with 80 MBq (n = 12) and 120 MBq (n = 11) of LNC^188^Re-SSS, sham rats (n = 12), blank LNC (n = 8) and 80 MBq of ^188^Re-perrhenate solution (n = 4).

As shown in Figure 2, all animals of the control groups died due to tumor progression at D105, with a median survival time of 51±19 days, 77±19 days, and 57±24 days for blank LNC, sham, and ^188^ReO_4_^-^ groups, respectively. There was no significant difference between the three control groups (*p*>0.05).

Following treatment with LNC^188^Re-SSS (80 MBq) therapeutic efficiency was demonstrated by an increase in the median survival from 54 to 107% as compared to control groups with up to 7/12 long-term survivors in the LNC^188^Re-SSS group (80 MBq). Even if a slight difference in median survival time was observed between LNC^188^Re-SSS – 80 MBq (118±27 days) and LNC^188^Re-SSS – 120 MBq (97±18 days), comparison with these two groups was not significant (p = 0.4431).

**Figure 3:**
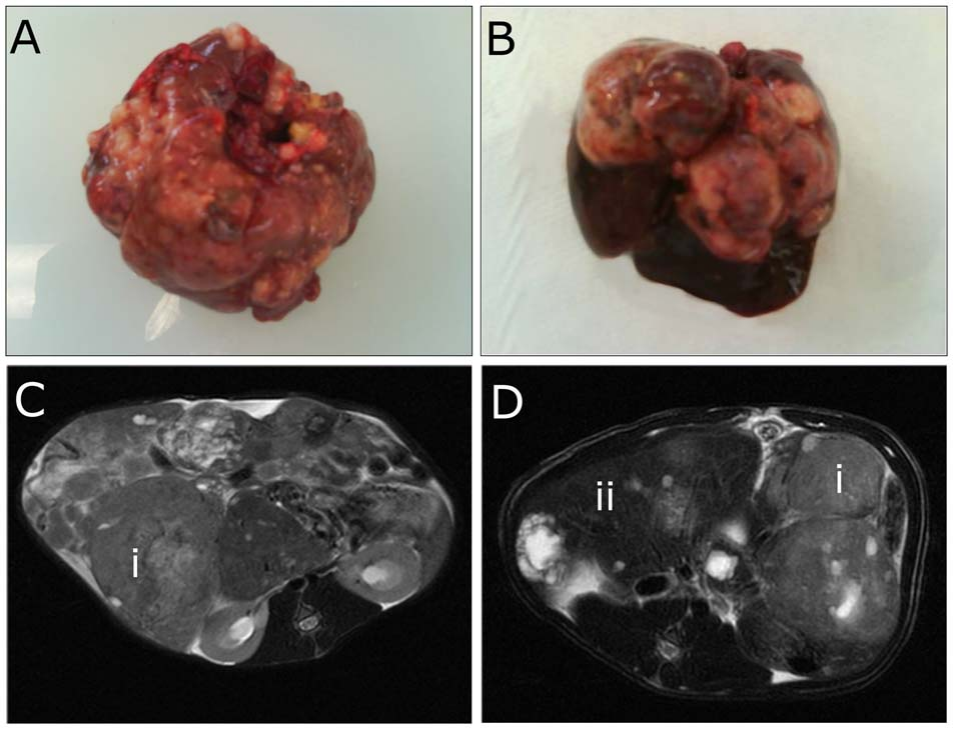
Tumor volume assessment by MRI. A and B represent macroscopic views at <D101 of control rats and LNC^188^Re-SSS rats, respectively; T2-weighted images of control rats (C) and LNC^188^Re-SSS (D) at D100 after the end of tumor induction. Tumors appear as hypersignals (i arrows); healthy liver (ii arrow).

Tumor volume assessment was performed using two different evaluation methods: MRI and macroscopic study. Figure 3 shows MRI and macroscopic views of control groups (sham, ^188^ReO_4_^-^ and blank LNC) and LNC^188^Re-SSS – 80 MBq rats at D100 (MRI) and D101 (macroscopy) after the end of tumor induction. Liver tumors represented approximately 100% of the liver parenchyma for control rats, while they occupied only 50% of the liver tissue for LNC^188^Re-SSS – 80 MBq rats. Therefore, 50% of healthy liver tissue appeared to be preserved following LNC^188^Re-SSS – 80 MBq treatment. This observation was confirmed by histopathological analysis (data not shown).

### Transaminases assessment

Transaminases were assessed in each group and compared to healthy rat data. As shown in Tables 3 and 4, the AST and ALT kinetics were very similar, with a slight liver toxicity starting at D12 for the LNC^188^Re-SSS– 80 MBq group (AST, LNC^188^Re-SSS – 80 MBq group: 104 UI/mL, sham group: 74 UI/mL, ^188^ReO_4_^-^ group: 87 UI/mL, blank LNC group: 84 UI/mL, healthy rats: 76 UI/mL), which faded with normalization of AST levels at D55 (LNC^188^Re-SSS– 80 MBq: 80 UI/mL, sham group: 95 UI/mL, ^188^ReO_4_^-^ group: 106 UI/mL, blank LNC: 93 UI/mL, healthy rats: 98 UI/mL) and at D90 for ALT levels (LNC^188^Re-SSS – 80 MBq: 78 UI/mL, sham group: 66 UI/mL, ^188^ReO_4_^-^ group: 78 UI/mL, blank LNC: 76 UI/mL, healthy rats: 87 UI/mL). On the other side, hepatotoxicity was highlighted after a single injection of 120 MBq of LNC^188^Re-SSS with ALT and AST levels higher in comparison with control groups at D12 (AST, LNC^188^Re-SSS – 120 MBq group: 151 UI/mL; ALT, LNC^188^Re-SSS – 120 MBq group: 146 UI/mL) up to D105 (AST, LNC^188^Re-SSS – 120 MBq group: 129 UI/mL; ALT, LNC^188^Re-SSS – 120 MBq group: 122 UI/mL).

**Table 3:** AST kinetics.

AST UI/mL						
	LNC^188^Re-SSSSGroup 1(80 MBq; n = 12)*p* values vs Group 6	LNC^188^Re-SSSSGroup 2(120 MBq; n = 11)*p* values vs Group 6	ShamGroup 3n = 12*p* values vs Group 6	^188^ReO_4_^-^Group 4n = 4*p* values vs Group 6	Blank LNCGroup 5n = 10*p* values vs Group 6	Healthy ratsGroup 6n = 10-
**d12**	104±7.260.421*	151±9.480.0283*	74±19.80.2547	87±11.310.1292	84±11.670.1361	76±11.78-
**d18**	97±8.120.0489*	147±14.620.0193*	77±16.320.3415	97±9.730.0418*	78±9.560.3190	82±9.45-
**d25**	102±10.760.0374*	143±10.780.0342	88±12.780.2172	92±10.720.0426*	95±15.740.0435*	79±10.32-
**d30**	114±11.850.0372*	140±15.040.0187*	85±18.590.7341	96±8.430.3517	106±22.160.1214	89±13.57-
**d45**	109±12.640.464*	129±3.110.0231*	105±20.10.1322	110±11.870.1212	108±25.90.1189	97±9.74-
**d55**	80±20.360.4522	127±6.80.0251*	95±16.620.3781	106±19.950.1429	93±11.430.5123	98±12.78-
**d65**	84±7.140.5631	126±4.890.0467	82±14.850.5515	95±12.380.4287	85±14.750.5744	109±21.32-
**d80**	87±10.890.1865	128±19.990.0359*	92±13.670.1432	88±10.240.3428	97±17.590.4682	89±10.38-
**d90**	77±5.920.5349	131±8.830.0321*	137±40.120.0287*	84±8.560.4864	79±7.640.5276	94±4.66-
**d105**	84±30.810.4326	129±10.650.0211*	188±85.870.0083**	--	--	103±23.61-
**d130**	91±9.790.1218	136±14.320.0107	--	--	--	85±8.4-
**d152**	159±57.980.0066**	--	--	--	--	76±27.40-

The determination of the AST content in plasma samples for LNC^188^Re-SSS (80 MBq and 120 MBq groups), sham, ^188^ReO_4_^-^, and blank LNC groups at D12, D18, D24, D25, D30, D45, D55, D65, D80, D90, D105, D130, and D152 after the end of tumor induction. Results are expressed in IU/mL of AST, mean ± SD. Comparisons of AST content versus healthy rats (*p<0.05; **p<0.01).

**Table 4:** ALT Kinetics.

ALT UI/mL						
	LNC^188^Re-SSSSGroup 1(80 MBq; n = 12)*p* values vs Group 6	LNC^188^Re-SSSSGroup 2(120 MBq; n = 11)*p* values vs Group 6	ShamGroup 3n = 12*p* values vs Group 6	^188^ReO_4_^-^Group 4n = 4*p* values vs Group 6	Blank LNCGroup 5n = 10*p* values vs Group 6	Healthy ratsGroup 6n = 10-
**d12**	95±8.720.0392*	146±9.780.0083**	64±10.010.1281	83±8.480.1042	74±9.120.2148	67±12.14-
**d18**	101±13.620.0261*	148±12.640.0021**	73±9.410.0021**	74±7.620.1118	68±12.560.3512	72±7.65-
**d25**	98±10.370.0437*	141±10.560.0092**	83±13.670.3163	69±11.740.0912	82±10.860.1006	87±10.29-
**d30**	87±14.670.1872	134±12.760.0017**	79±10.980.4113	84±8.940.2275	79±9.670.3127	76±8.34-
**d45**	99±14.140.0439*	128±6.780.0376*	83±22.050.1024	93±11.570.3510	95±20.110.3121	86±11.33-
**d55**	88±12.630.1132	133±13.950.0051**	76±6.510.2941	67±7.840.2230	77±7.120.1949	80±9.52-
**d65**	90±15.650.0734	126±12.560.0421*	79±8.940.4376	72±5.780.3311	81±9.530.2414	84±14.48-
**d80**	73±12.840.5635	125±24.290.0034**	72±7.360.2037	84±9.230.1882	92±15.670.0613	79±7.48-
**d90**	78±0.740.4523	129±10.850.0288*	66±9.950.4579	78±10.450.1776	76±6.890.2430	87±15.03-
**d105**	86±24.740.2649	122±11.430.0107*	120±14.810.0119*	--	--	77±5.02-
**d130**	74±11.650.3498	139±9.520.0034**	--	--	--	69±10.61-
**d152**	123±27.980.0056**	--	--	--	--	73±12.59-

The determination of the ALT content in plasma samples for LNC^188^Re-SSS (80 MBq and 120 MBq groups), sham, ^188^ReO_4_^-^, and blank LNC groups at D12, D18, D24, D25, D30, D45, D55, D65, D80, D90, D105, D130, and D152 after the end of tumor induction. Results are expressed in IU/mL of AST, mean ± SD. Comparisons of ALT content versus healthy rats (*p<0.05; **p<0.01).

### VEGF quantification

**Figure 4:**
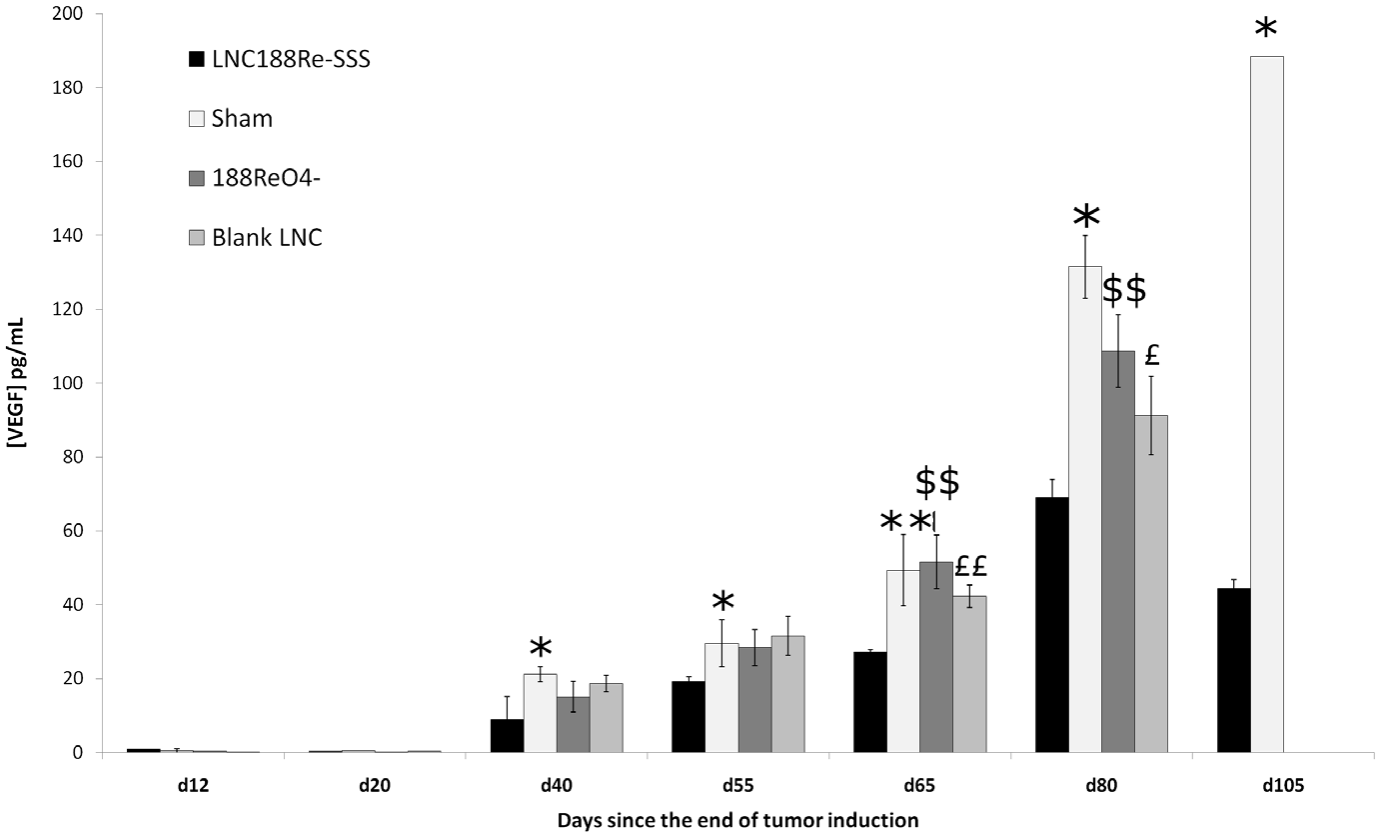
Determination of the VEGF content in plasma samples. The concentration of VEGF for each group. Results are expressed in pg/mL of VEGF, mean ± SD; comparison of the VEGF content in the LNC^188^Re-SSS versus the sham group (*p<0.05; **p<0.01), ^188^ReO_4_^-^ group (^$$^p<0.01), and blank LNC (^£^p<0.05; ^££^p<0.01).

Results showed major differences between LNC^188^Re-SSS – 80 MBq and the control groups (Figure 4). At D65, the VEGF concentration was approximately half as high in LNC^188^Re-SSS rats versus each control group (LNC^188^Re-SSS – 80 MBq group: 21.27 pg/mL; sham rats: 49.31 pg/mL; ^188^ReO_4_^-^ group: 51.57 pg/mL; blank LNC group: 42.28 pg/mL) (p_LNC188Re-SSS/sham_ = 0.0083; p_LNC188Re-SSS/blank LNC_ = 0.016; p_LNC188Re-SSS/188ReO4-_ = 0.0008). This remains lower up to D80 (LNC^188^Re-SSS – 80 MBq group: 69 pg/mL; sham rats: 131.50 pg/mL; ^188^ReO_4_^-^ group: 108.7 pg/mL; blank LNC group: 91.21 pg/mL) (p_LNC188Re-SSS/sham_ = 0.043; p_LNC188Re-SSS/blank LNC_ = 0.0188; p_LNC188Re-SSS/188ReO4-_ = 0.0023).

## Discussion

The use of LNCs, with a structure similar to lipoproteins, could represent a promising therapeutic modality for HCC management, as they modify the biodistribution of entrapped therapeutic agents [12], [13], [14]. These nano-objects include only FDA-approved excipients and are composed of a lipidic core leading to the entrapment of lipophilic molecules such as ^188^Re-SSS [12], surrounded by a tension-active shell which induce physico-chemical properties different from those of the drug. Additionally, their nanometric scale (LNC^188^Re-SSS mean diameter: 50 nm), and their low polydispersity index but also, the reduced viscosity of the drug, represent real advantages. It could likely avoid the excessive embolization process observed with chemoembolization (DC-beads™ – mean diameter: 300–500 µm [15]) and may penetrate more deeply inside the tumor blood vessels in comparison with ^90^Y-microspheres devices (mean diameter around: 20–40 µm).

The first step in this study was to demonstrate the relevance of the encapsulation of Rhenium-188 for selective internal radiotherapy on a HCC rat model. The observed liver uptake following LNC^188^Re-SSS injection and ^188^Re-perrhenate accumulation in the stomach, which have been already reported in the literature [13], [14], validate the interest of the encapsulation of Rhenium-188. Organ biodistribution results indicated that LNC^188^Re-SSS clearance from the blood was mainly ascribed to the liver. The enhancement permeability retention effect (EPR) may account for these findings [6]. This phenomenon could be explained by the size of LNC^188^Re-SSS but Küpffer cells could be another explanation for these observations. In fact, it has been demonstrated that Küpffer cells and some macrophages are involved in nanoparticle capture [16]. Thus, the physico-chemical properties of particulate systems improve the liver uptake of Rhenium-188.

The internal radiotherapy study by intra-arterial injection of LNC^188^Re-SSS was carried out on a chemically induced (DENA) HCC rat model. 100% tumor take was observed, with a median survival time ranging from 51 to 77 days after the end of tumor. Three control groups were performed: ^188^Re-perrhenate, blank LNC and sham groups. Neither the injection of the ^188^Re-perrhenate solution nor the injection of blank LNC significantly modified rat survival, as the median survival time was 58 and 51 days respectively. The median survival time of the sham group (77 days) was higher than that of the ^188^Re-perrhenate and blank LNC groups. This could be explained by a less invasive surgery, as neither hepatic artery catheterization nor injection, which could modify the hemodynamic parameters, were performed. As a consequence, treated groups (LNC^188^Re-SSS – 80 MBq; LNC^188^Re-SSS – 120 MBq) were compared with both ^188^Re-perrhenate or blank LNC groups and sham group.

The LNC^188^Re-SSS – 80 MBq treatment was the most effective with 7/12 or 2/12 rats being long-term survivors and an IMST ranging from 107% to 54% according to the control group (^188^Re-perrhenate groups or sham, respectively). These results correlated with MR images and macroscopic views, demonstrating a slowdown in tumor progression in the LNC^188^Re-SSS – 80 MBq group.

Increasing the absorded dose to the liver has shown no interest in terms of survival efficacy as LNC^188^Re-SSS – 120 MBq group gave rise to the worse increase in median survival (IMST% ranging from 26% to 70% according to the control group). Hepatotoxicity demonstrated by higher levels of transaminases, could explain its less efficiency in term of survival.

Angiogenesis plays a key role in the pathogenesis of many cancers [17]. HCC, a hypervascular tumor, is mainly supplied by hepatic artery, whereas normal liver parenchyma and dysplastic nodules are largely supplied by the portal vein [18]. HCCs have been shown to express many angiogenic factors including VEGF [19]. Moreover, VEGF expression by the tumor and VEGF levels in patients' blood have been shown to correlate with the size, invasiveness, metastases, and prognosis of HCC [20]. As a consequence, the assessment of VEGF concentrations in plasma samples was carried out in each group except for LNC^188^Re-SSS – 120 MBq group, which demonstrated worse results in terms of survival and hepatotoxicity. The lower VEGF levels, in LNC^188^Re-SSS – 80 MBq group in comparison with control groups, could reflected an altered angiogenesis in the rat HCC model, which could reduced tumor progression confirmed by MRI.

Our results pointed out the advantage in using lipid nanocapsules, a drug delivery system for SIRT. In that field, an important breakthrough for HCC treatment has been done, with drug eluting beads (DEB, DC-beads™), ^90^Y-microspheres (TheraSphere®, SIR-Sphere®), ^188^Re-microspheres and nanocarriers. Dhanasekaran *et al*. demonstrated that doxorubicin DEB therapy with unresectable HCC provides a survival advantage over treatment with conventional chemoembolization [21]. However, micrometric size of DC-beads (300 to 500 µm) could generate excessive embolisation leading to Post-Embolization Syndrome (PES) [22]. Meanwhile DC-beads, SIRT with ^90^Y-microspheres is another palliative HCC treatment option, with up to 50% in tumor HCC size reduction [23], [24], [25], [26]. ^188^Re-microspheres were also developed for HCC treatment with decrease in tumor growth after ^188^Re-microsphere injection [27], [28].

Nanocarriers [29], [30] enable to load chemotherapeutic agents such as docetaxel [31] or oligonucleotides [32] were developed in order to reduce PES observed with DC-beads. Results demonstrated a slowdown in tumor progression but most of these preclinical studies assessed their efficacy on a subcutaneous HCC model.

We have developed 50 nm-lipid nanocapsules loaded with Rhenium-188 for selective internal radiotherapy and have assessed their efficiency on a chemically induced HCC rat model, known for its physiological properties similar to human hepatocarcinoma. Due to their nanometric scale, no embolisation process was possible. This could represent a real advantage as tumoral hypoxia areas could be reduced allowing a better efficiency of ionizing radiations.

Recently, sorafenib, an inhibitor of the VEGF receptor, has been shown to prolong the median survival time by 3 months in patients with advanced HCC [33]. As LNC^188^Re-SSS seems to alter angiogenic process, their combination with sorafenib as an adjuvant therapy could be a valuable approach in the treatment of advanced HCC.

In conclusion, ^188^Re-loaded LNCs appear to be an encouraging new radiopharmaceutical carrier for HCC internal radiotherapy which could penetrate more deeply inside the tumor blood vessels. A comparative study of LNC^188^Re-SSS and ^90^Y-microspheres SIRT, already used in clinical application, will probably provide informations of the effect of the size of particle systems and also in term of dosimetry.
